# Implications of m6A-associated snRNAs in the prognosis and immunotherapeutic responses of hepatocellular carcinoma

**DOI:** 10.3389/fimmu.2022.1001506

**Published:** 2022-11-02

**Authors:** Cheng Zhang, Wangjian Zhang, Yongjie Shui, Ping Li, Zhifeng Tian, Shiwei Duan, Qichun Wei

**Affiliations:** ^1^ Department of Medical Oncology, The Affiliated Hospital of Hangzhou Normal University, Hangzhou, Zhejiang, China; ^2^ Department of Radiation Oncology, The Second Affiliated Hospital, Zhejiang University School of Medicine, Hangzhou, Zhejiang, China; ^3^ Key Laboratory of Cancer Prevention and Intervention, China National Ministry of Education, Zhejiang University School of Medicine, Hangzhou, Zhejiang, China; ^4^ School of Public Health, Sun Yat-sen University, Guangzhou, Guangdong, China; ^5^ School of Medicine, Zhejiang University City College, Hangzhou, Zhejiang, China

**Keywords:** m6A, snRNA, hepatocellular carcinoma, prognosis, immunotherapy

## Abstract

**Background:**

Hepatocellular carcinoma (HCC) is the most prevalent pathological type of liver cancer worldwide with high mortality and poor prognosis. N6-methyladenosine (m6A) can modify RNAs such as mRNA, lncRNA, miRNA, and tRNA, thereby playing a critical role in the pathogenesis of HCC. However, the role of m6A-associated small nuclear RNA (snRNA) in the prognostic value and immunotherapeutic response in HCC remains unclear.

**Materials and methods:**

In this study, snRNA expression data, gene mutation data, and clinical data of HCC patients were acquired from The Cancer Genome Atlas (TCGA) database. We used the least absolute shrinkage and selection operator (LASSO) Cox regression analysis to identify significant prognostic m6A-associated snRNAs, and then developed a multivariate Cox model based on the selected snRNAs. HCC patients were split into low- and high-risk groups based on the median risk score. We subsequently performed Kaplan-Meier curve analysis to estimate overall survival (OS) by clinicopathological characteristics and tumor mutational burden (TMB) status in low- and high-risk HCC patients. Finally, we compared the immunotherapeutic response as represented by tumor immune dysfunction and exclusion (TIDE) scores between the two risk groups.

**Results:**

Eight m6A-associated snRNAs were selected as independent predictors to develop the risk model. Our results revealed that the OS of HCC patients in the high-risk group was significantly worse than that in the low-risk group on clinicopathologic characteristics, including age (≤65 years and >65 years), gender (male), grade (G I-II and G III-IV) and TNM staging (Stage I-II and Stage III-IV). In addition, the OS of low-TMB and low-risk group was longer than that of high-TMB and high-risk group. The TIDE score indicated that HCC patients in the high-risk group were more susceptible to immunotherapy.

**Conclusion:**

Our study suggests that m6A-associated snRNAs may be useful biomarkers for the prognosis of HCC and that m6A-associated snRNA models can predict the effect of immunotherapy in HCC patients.

## Introduction

Primary liver cancer (PLC) is a prevalent malignant tumor, ranking sixth in incidence and third in mortality among all types of tumors in the world (1). Hepatocellular carcinoma (HCC), the main pathological type of PLC, develops from chronic hepatitis and cirrhosis through a series of pathophysiological processes (2, 3). Hepatocarcinogenesis is associated with many risk factors, such as hepatitis virus infection, alcohol addiction, dietary toxin exposure, and genetic aberrations (4–7). Current treatments for HCC include hepatectomy, chemotherapy, radiotherapy, targeted therapy, immunotherapy, and liver transplantation (8, 9). However, more than 70% of patients with advanced HCC usually obtain limited therapeutic benefits. Most HCC patients may experience recurrence or distant metastasis after first-line therapy (10). Thus, clarifying the molecular mechanisms of HCC pathogenesis and exploring new targets are crucial for the diagnosis and therapy of HCC.

m6A is the most important internal modification of RNA epitranscriptomes in eukaryotes (11). m6A-mediated RNAs, including mRNA, rRNA, tRNA, microRNA (miRNA), long noncoding RNA (lncRNA), and small nuclear RNA (snRNA), play imperative roles in many cellular processes through post-transcriptional regulation of gene expression (12). m6A methylation process is dynamic and reversible. The biological activity is balanced by methyltransferases to add m6A modifications (writers), demethylases to remove m6A (erasers), and m6A-binding proteins to recognize m6A (readers) (13, 14). Dysregulation in writers, erasers, and readers can lead to many diseases, such as HCC. For example, writer METTL3 was associated with poor prognosis in HCC. Decreased METTL3 could reduce HCC cell proliferation and migration, and suppress HCC tumorigenicity and lung metastasis (15). In addition, eraser FTO could demethylate pyruvate kinase M2 (PKM2) mRNA and enhance translation. Knockdown of FTO inhibited HCC cell proliferation and induced G0/G1 phase arrest (16). However, the comprehensive molecular mechanism of m6A-mediated RNA in HCC pathogenesis remains unclear.

Small nuclear RNAs are non-coding RNAs of approximately 150 nucleotides which are commonly observed in the splicing speckles and Cajal bodies in eukaryotic cells (17). In the nucleus, snRNAs may splice pre-messenger RNAs, mediate transcription factors, and regulate gene expressions (18, 19). snRNA always binds to some specific proteins to form a complex called small nuclear ribonucleoprotein (snRNP), mainly composed of U1, U2, U4, U5, and U6 spliceosomal RNAs (20). Recent studies found that m6A-modified snRNAs may affect RNA biogenesis, and play a vital role in tumorigenesis and cancer progression (21, 22). For example, m6A modification of U6 snRNA could be catalyzed by the methyltransferase METTL16 and removed by the demethylase FTO (23–26). U6 snRNA was overexpressed in breast cancer and cervical carcinoma (27, 28), which was useful for the diagnosis, treatment, and prognosis of these cancers. Nevertheless, the biological function of m6A-mediated snRNAs in the pathogenesis of HCC remains ambiguous.

In this study, we aimed to explore the role of m6A-associated snRNAs in predicting the prognosis and immunotherapeutic response of HCC patients. We developed a risk model based on m6A-associated snRNA to analyze the OS of HCC patients on clinicopathologic characteristics (including age, gender, grade, and TNM staging) and TMB. We also used TIDE scores to analyze the effect of immunotherapy based on the m6A-associated snRNA risk model. Our study may be helpful for the guidance of personalized immunotherapy for HCC patients.

## Materials and methods

### Data acquisition and processing

We obtained RNA-seq transcriptomic data, clinical information (including age, gender, grade, TNM staging, survival time and survival status) (**Table 1**), and gene mutation data of HCC patients from TCGA database on June 15, 2022. A total of 424 samples were obtained from TCGA, including 374 tumor tissues and 50 non-tumor tissues. The RNA-sequencing data files were merged into one RNA matrix file by a Perl script. The RNA-matrix was converted into an array of gene symbols for further analysis. Using the GRCh38 annotation file downloaded from the GENCODE database, 1872 snRNAs and 19573 mRNAs were identified based on the gene symbols. R package edgeR was applied to assess differentially expressed snRNAs and differentially expressed mRNAs between tumor and non-tumor tissues for further analysis. Thresholds were set for |log_2_ (fold change) | > 1 and adjusted p < 0.05. Since all of these data from TCGA are public, no ethics committee approval is required.

**Table 1:** Clinical profiles of HCC patients from TCGA dataset.

Characteristics	Subgroup	Patients (n)
Gender	Male	255
	Female	122
Age (years)	≤65	235
	>65	141
	Unkonwn	1
Grade	G1	55
	G2	180
	G3	124
	G4	13
	Unknown	5
T stage	T1	185
	T2	95
	T3	81
	T4	13
	TX	1
	Unknown	2
N stage	N0	257
	N1	4
	NX	115
	Unkonwn	1
M stage	M0	272
	M1	4
	MX	101
Pathological TNM staging	Stage I	175
	Stage II	87
	Stage III	86
	Stage IV	5
	Unknown	24
Survival status	Alive	245
	Dead	132

### Selection of m6A genes and m6A-associated snRNAs

We obtained expression profiles of 23 m6A regulators from transcriptome data. 95 m6A-associated snRNAs were identified by the Pearson’s correlation test with the standard of Pearson cor > 0.1 and *P* < 0.05. Then, the R package “ggalluvial” was used to visualize the association network between 23 m6A-regulator genes and snRNAs.

### Establishment and validation of m6A-associated snRNA model

To identify potentially optimal m6A-associated snRNAs, we randomly split HCC patients from TCGA database into training and testing datasets in a 1:1 ratio. First, nine significant prognostic m6A-associated snRNAs were filtered out by univariate Cox regression analysis. Then, Least Absolute Shrinkage and Selection Operator (LASSO) Cox regression analysis was employed to confirm the nine significant prognostic m6A-associated snRNAs with penalty parameters estimated by 1000-fold cross-validation. Finally, multivariate Cox regression analysis was conducted to establish a risk model based on eight m6A-associated snRNAs which were selected from the nine significant prognostic snRNAs in this process. Risk score was computed by the following method: Risk score =Σn i=1 coef_i_ × expr_i_, where coef_i_ indicated the coefficient of the corresponding m6A-associated snRNA, and expr_i_ represented the expression level of the m6A-associated snRNA. According to the median risk score, the HCC patients were split into high- and low-risk groups. Subsequently, principal component analysis (PCA) was conducted to observe the distributions of the low- and high-risk groups. The distribution of risk scores, survival status and heatmaps of m6A-associated snRNAs were evaluated in the training and testing datasets.

### Independent prognosis analysis of m6A-associated snRNA model

Univariate and multivariate Cox regression analyses were used to assess the accuracy of prognostic m6A-associated snRNA signatures as independent risk factors compared with other clinicopathological characteristics (including age, gender, grade and TNM staging) in HCC patients. Receiver operating characteristic (ROC) curves were constructed to evaluate the accuracy of m6A-associated snRNA models in predicting HCC prognosis, and the area under the curve (AUC) was calculated.

### Kaplan-Meier survival analysis

Kaplan-Meier survival analysis was performed to assess OS based on clinicopathological characteristics in low- and high-risk HCC patients by using the R package “survMiner”. A matched comparison was conducted on clinicopathological characteristics, including age (≤65 years and >65 years), gender (male and female), grade (G I-II and G III-IV), and TNM staging (Stage I-II and Stage III-IV). A *P* value less than 0.05 indicated a statistical significance.

We applied the R package “maftools” to evaluate gene mutation data in low- and high-risk HCC patients. TMB was evaluated according to tumor-specific mutated genes. Kaplan-Meier curve analysis was conducted to assess OS in HCC patients based on TMB status. A *P* value less than 0.05 indicated a statistical significance.

### Functional analysis

We conducted Gene Ontology (GO) functional enrichment analysis (including biological processes, cellular components, and molecular functions) to explore the potential signaling pathways of m6A-associated snRNAs in HCC. R package “clusterProfiler” was utilized to perform this analysis. Subsequently, significant GO terms were visualized in a bar graph by using “enrichplot” package. In addition, we explored the immune functions in HCC patients based on m6A-associated snRNA model, and distinguished the difference in immune activities and functions between low- and high-risk HCC patients. This process utilized the R package “pheatmap”. A *P* value less than 0.05 indicated a statistical significance.

### Exploration of m6A-associated snRNA models in immunotherapeutic response

Tumor Immune Dysfunction and Exclusion (TIDE) is a computational framework for assessing the potential of tumor immune escape (29). A low TIDE score means weak potentiality of tumor immune escape, and while these patients may exhibit a strong immunotherapeutic response. In this study, we conducted TIDE algorithm based on m6A-associated snRNA model to predict immunotherapeutic response in HCC patients. A *P* value less than 0.05 indicated a statistical significance.

## Results

### Identification of m6A-associated snRNAs in HCC patients

**Figure 1:**
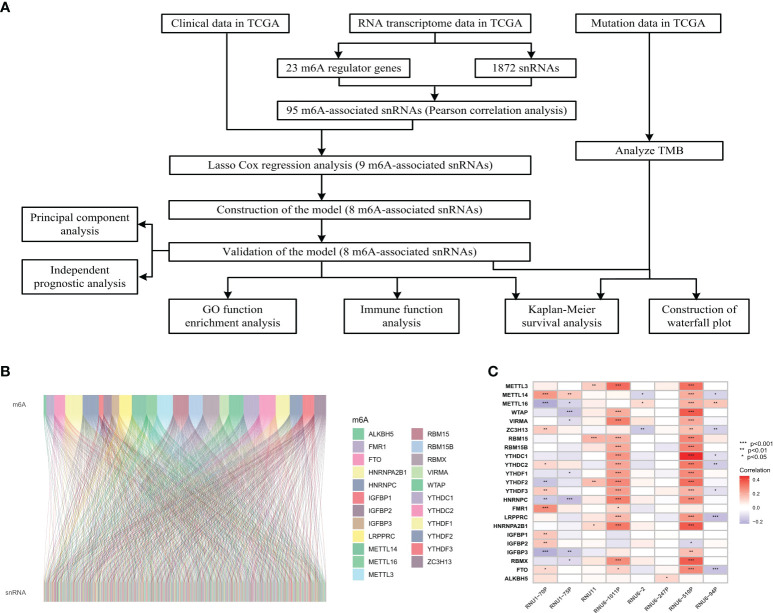
Flowchart and association analysis between m6A regulators and snRNAs. **(A)** Flowchart of this study. **(B)** Association between m6A RNA regulators and snRNAs. **(C)** Heatmap of prognostic snRNAs associated with m6A RNA regulators.

The flow diagram of this study was shown in **Figure 1A**. The expression matrix of 23 m6A regulator genes and 1872 snRNAs were abstracted from TCGA database. The association network of m6A regulators and snRNAs was visualized in **Figure 1B**, and 95 m6A-associated snRNAs were selected in this study. The associations between 23 m6A regulator genes and eight significant prognostic snRNAs were shown in **Figure 1C**, which showed that RNU6-510P had significant associations with 20 m6A regulator genes, while RNU6-247P had a significant association with one m6A regulator gene (ALKBH5).

### Construction and validation of m6A-associated snRNA risk model

**Figure 2:**
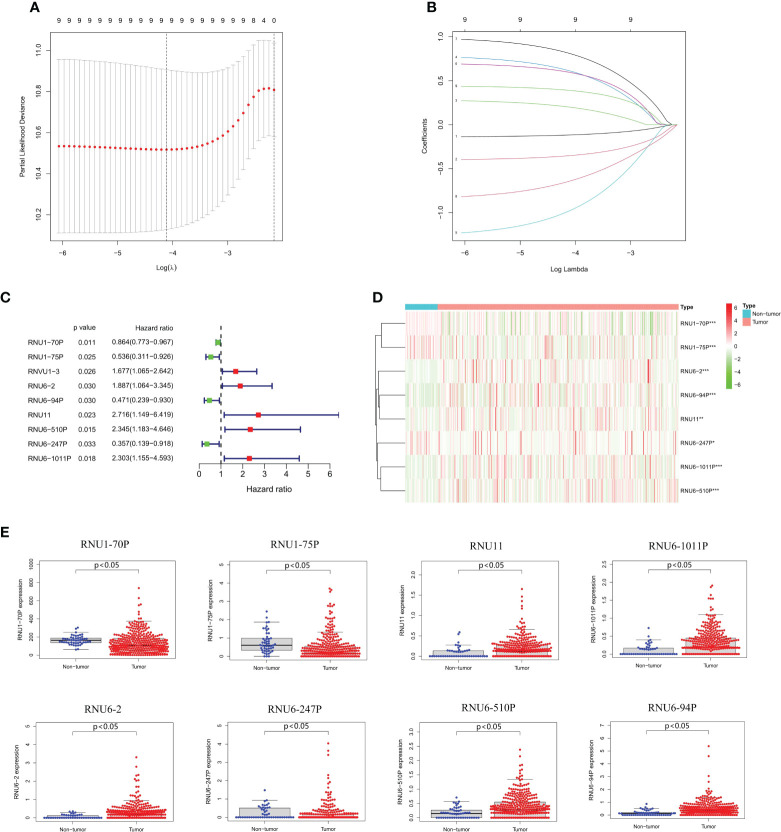
Construction of a risk model based on m6A-associated snRNAs. **(A)** Lasso Cox regression analysis of prognostic snRNA associated with m6A RNA regulators. **(B)** Cross-validation of parameter tuning and selection in the LASSO model. **(C)** Forest plot of predictive prognostic snRNAs associated with m6A RNA regulators. **(D)** Heatmap of eight m6A-associated snRNA expression. **(E)** Expression of eight m6A-associated snRNAs between non-tumor and tumor tissues.

**Figure 3:**
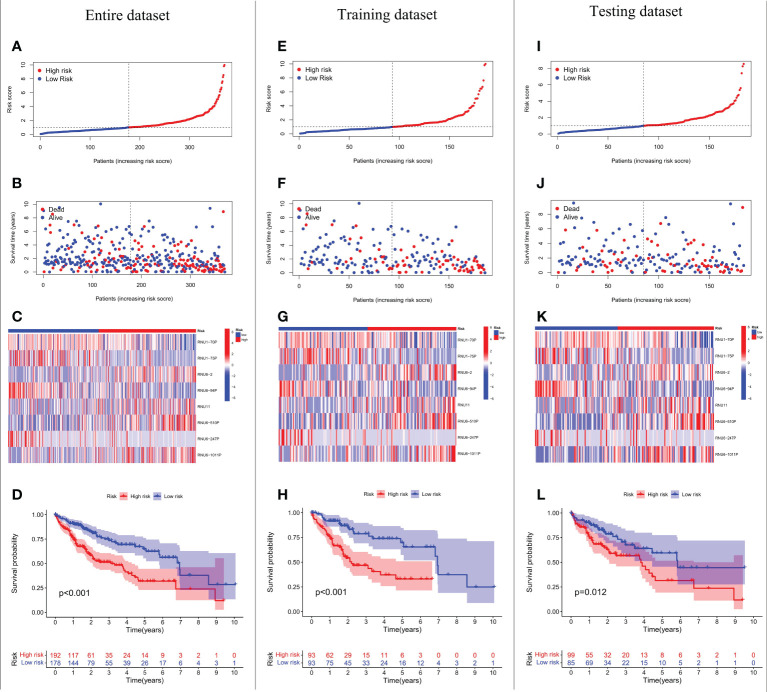
Prognostic value of the risk model based on eight m6A-associated snRNAs. Risk score distribution of HCC patients in the entire dataset **(A)**, the training dataset **(E)**, and the testing dataset **(I)**. Distribution of survival status of HCC patients in the entire dataset **(B)**, the training dataset **(F)**, and the testing dataset **(J)**. Heatmap of prognostic m6A-associated snRNA expression in HCC patients in the entire dataset **(C)**, the training dataset **(G)**, and the testing dataset **(K)**. OS of HCC patients based on risk scores in the entire dataset **(D)**, the training dataset **(H)**, and the testing dataset **(L)**.

We conducted LASSO-Cox regression analysis to identify nine significant prognostic snRNAs selected from 95 m6A-associated snRNAs to construct risk scores for the prediction of prognosis in HCC patients (**Figures 2A–C**). Then we conducted multivariate Cox regression analysis to establish the risk model based on eight m6A-associated snRNAs which were selected from the nine significant prognostic snRNAs in this process. The expression of eight m6A-associated snRNAs was obviously higher in tumor tissues than in non-tumor tissues (**Figures 2D, E**). HCC patients were divided into low- and high-risk groups according to the median risk score. The distribution of risk scores for the entire dataset between low-risk and high-risk groups was shown in **Figure 3A**. **Figure 3B** showed that HCC patients with higher risk scores had shorter survival times than those with lower risk scores. The relative expression of the eight m6A-associated snRNAs in the entire dataset was shown in **Figure 3C**. Survival analysis indicated that OS was longer in the low-risk group than in the high-risk group (p < 0.001) (**Figure 3D**). To verify the accuracy of the m6A-associated snRNA model in predicting HCC prognosis, we divided HCC patients into training and testing datasets. **Figures 3E–L** showed the distribution of risk scores and survival statuses, the expression of the m6A-associated snRNAs, and the OS in the training and testing datasets, indicating that the OS of HCC patients in the high-risk group was worse than that in the low-risk group (**Figures 3H, L**).

### PCA verifies the grouping capability of the m6A-associated snRNA model

**Figure 4:**
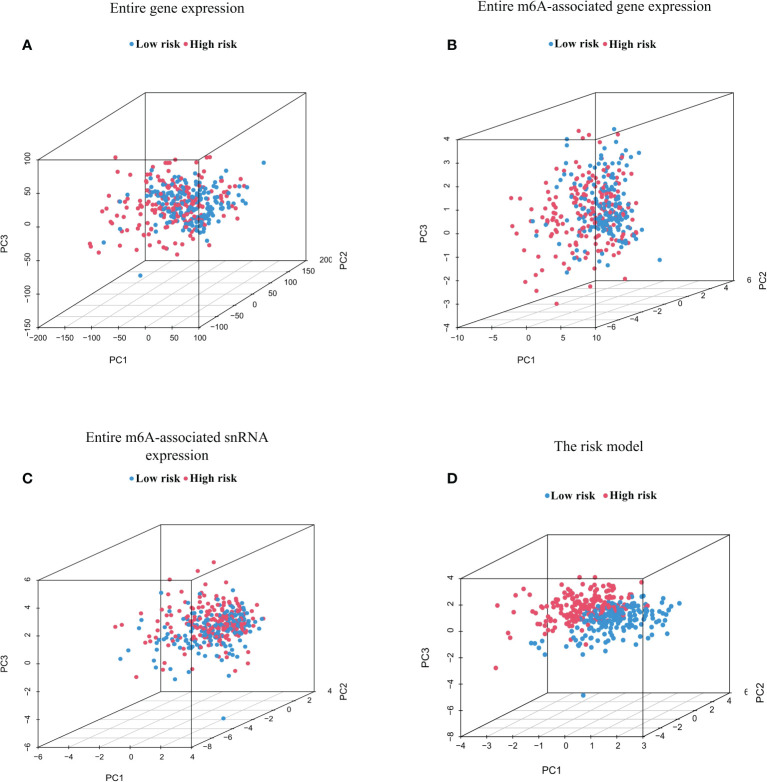
Principal component analysis (PCA) between high- and low-risk groups based on m6A-associated snRNAs. PCA between high- and low-risk groups based on the entire gene expression profiles **(A)**, the entire m6A-associated gene expression profiles **(B)**, the entire m6A-associated snRNA expression profiles **(C)**, and the risk model of eight m6A-associated snRNAs **(D)**.

PCA was conducted to test for different distributions between low-risk and high-risk groups in terms of the entire genes, 23 m6A regulator genes, 95 m6A-associated snRNAs, and the risk model constructed from eight m6A-associated snRNAs (**Figures 4A, D**). **Figures 4A–C** showed that distributions of high-risk and low-risk groups were relatively dispersed. However, the results based on the m6A-associated snRNA model indicated that the low-risk and high-risk groups had obviously different distributions (**Figure 4D**).

### Independent prognosis analysis of the risk model

**Figure 5:**
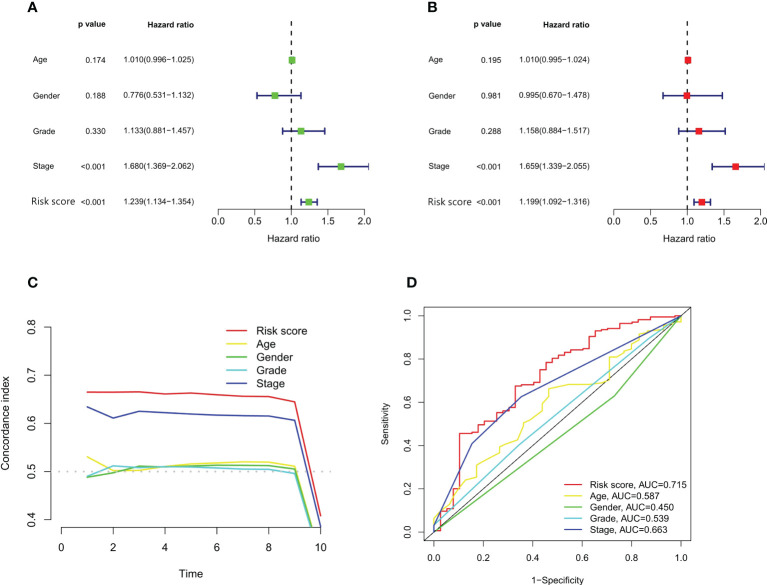
Independent prognostic analysis of the risk model. Forest plots of univariate **(A)** and multivariate **(B)** Cox regression analyses of the risk score and clinical characteristics. Concordance indexes **(C)** and ROC curve analysis **(D)** of the risk score and clinical characteristics.

We performed univariate and multivariate Cox regression analyses to assess whether m6A-associated snRNA models could independently predict prognosis in HCC. In univariate Cox regression analysis, the HR for the risk score and 95% confidence interval (CI) were 1.239 and 1.134–1.354 (p < 0.001), respectively (**Figure 5A**). In multivariate Cox regression analysis, the HR was 1.199 and 95% CI was 1.092–1.316 (p <0.001) (**Figure 5B**), indicating that the m6A-associated snRNA risk model was independent compared with some clinicopathological characteristics, such as age, gender, grade, and TNM staging. On the other hand, the concordance index of the m6A-associated snRNA risk score was superior to other clinicopathological characteristics, such as age, gender, grade, and TNM staging, indicating that the risk score could predict the prognosis of HCC (**Figure 5C**). Besides, the AUC value on the risk score was also higher than other clinicopathological features, indicating that the m6A-associated snRNA risk model for HCC was reliable (**Figure 5D**).

### The OS analysis of the low-risk and high-risk HCC patients on clinicopathologic characteristics and TMB status

**Figure 6:**
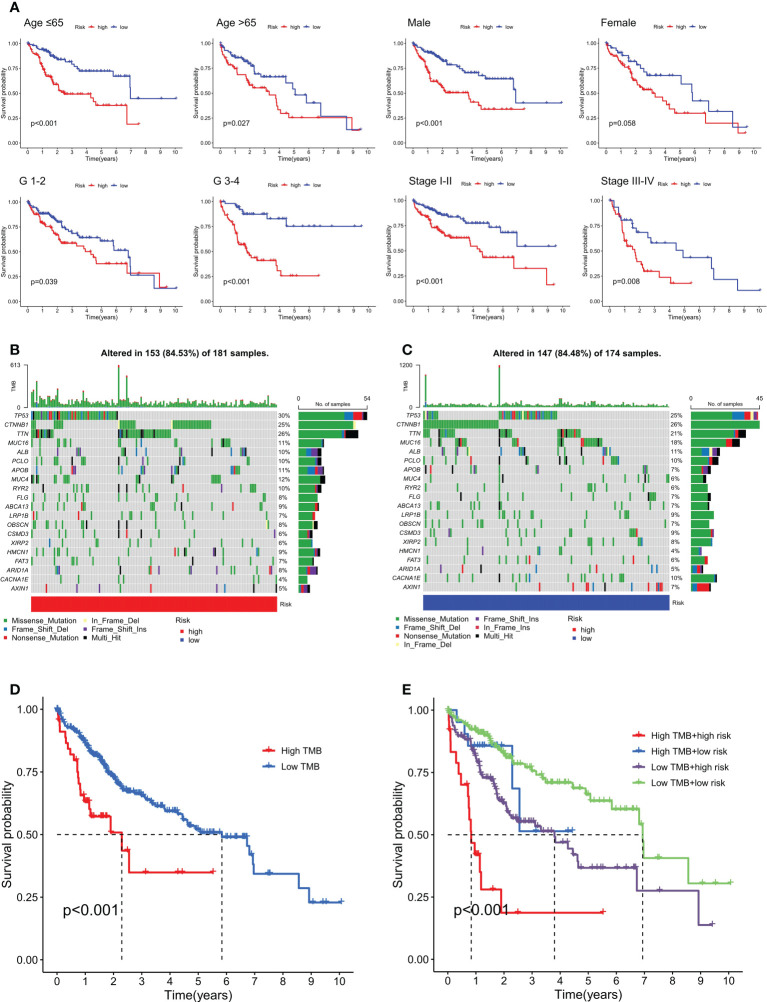
Kaplan-Meier curve analysis of OS in the low- and high-risk HCC patients on clinicopathological characteristics and TMB status. **(A)** Kaplan-Meier curve analysis of OS in the low- and high-risk HCC patients on clinicopathological characteristics (including age, gender, grade, and TNM staging). Waterfall plot of genes with high mutation frequencies in the high-risk group **(B)** and the low-risk group **(C)**. Kaplan-Meier curve analysis of OS based on TMB status **(D)** and both TMB status and risk scores **(E)**.

We analyzed the OS of low- and high-risk HCC patients based on clinicopathologic characteristics, including age, gender, grade, and TNM staging. Our findings indicated that high-risk HCC patients had poorer OS than low-risk HCC patients in terms of clinicopathological characteristics, including age (≤65 years and >65 years), gender (male), grade (G I-II and G III-IV), and TNM staging (Stage I-II and Stage III-IV). However, female high-risk HCC patients did not have significantly poorer OS than those low-risk HCC patients (**Figure 6A**). Furthermore, we conducted a subgroup OS analysis by clinicopathological characteristics (including age, grade, and TNM staging) to elucidate the difference between female and male. In female HCC patients, our results showed that high-risk group (G III-IV) had significantly poorer OS than that of low-risk group (**Supplementary Figure 1**). However, female high-risk HCC patients did not have significantly poorer OS than female low-risk HCC patients in terms of other clinicopathological characteristics, including age (≤65 years and >65 years), grade (G I-II), and TNM staging (Stage I-II and Stage III-IV) (**Supplementary Figure 1**). In male HCC patients, our results showed that high-risk group had significantly poorer OS than low-risk group in terms of some clinicopathological characteristics, including age (≤65 years and >65 years), grade (G III-IV), and TNM staging (Stage I-II) (**Supplementary Figure 2**). However, male high-risk HCC patients (G I-II and Stage III-IV) did not have significantly poorer OS than male low-risk HCC patients (**Supplementary Figure 2**).

Also, we analyzed the OS of high- and low-risk HCC patients on TMB status. The top 20 mutated genes with the highest rate of alterations in the high- and low-risk groups were displayed in **Figures 6B, C**, showing that 84.53% and 84.48% of HCC samples in the high-risk and low-risk groups were genetically altered, respectively. In addition, the low-TMB and low-risk groups had better OS than the high-TMB and high-risk groups (**Figures 6D, E**).

### Functional analysis and estimation of immunotherapeutic response

**Figure 7:**
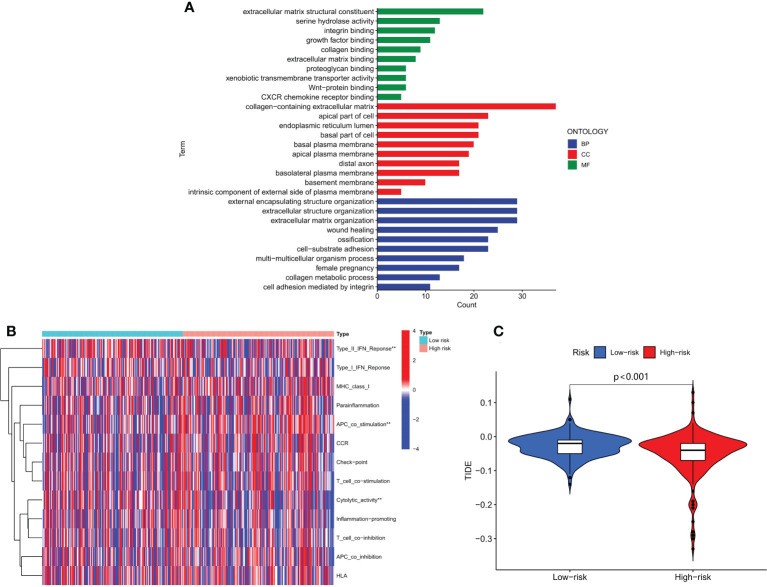
Functional analysis and estimation of immunotherapeutic response based on the m6A-associated snRNA model. **(A)** GO enrichment analysis based on the m6A-associated snRNA model. **(B)** Heatmap of the indicated standards of the immunity index based on the m6A-associated snRNA model. **(C)** TIDE scores in high- and low-risk HCC patients.

We conducted GO enrichment analysis to investigate molecular biological mechanisms of m6A-associated snRNAs in HCC. Our results showed that m6A-associated snRNAs played important roles in the pathogenesis of HCC through several biological processes, cellular components, and molecular functions, such as collagen metabolic process, basal plasma membrane, and serine hydrolase activity (**Figure 7A**). We then analyzed the biological activities and functions of several immune cells in HCC through the m6A-associated snRNA risk model. Our results indicated that the low-risk and high-risk groups had significant differences in certain immune functions, such as type II IFN response, APC co-stimulation, and cytolytic activity (**Figure 7B**). Subsequently, we explored the role of the m6A-associated snRNA model in immunotherapeutic response in HCC patients using the TIDE algorithm. Our results indicated that the TIDE score in the high-risk group was significantly lower than in the low-risk group, revealing that high-risk HCC patients may be more susceptible to immunotherapy (**Figure 7C**).

## Discussion

HCC is the main pathological type of liver cancer, with high mortality and poor prognosis. Numerous studies have focused on the occurrence, development, and treatment of HCC, and some studies have found that ncRNAs could predict the survival and immunotherapeutic response of HCC patients (30, 31). snRNA is a class of ncRNAs in the nucleus, which may splice pre-messenger RNAs, mediate transcription factors, and regulate gene expression (18, 19). Some studies showed that snRNAs could be involved in the carcinogenesis process of various cancers, including HCC. For example, Ding Y et al. revealed that the expression of snRNA RNU5E-1 was lower in HCC tissues than in adjacent tissues (32). In addition, snRNA RNU5E-1 was associated with tumor size, vascular tumor thrombus, differentiation degree, TNM staging, tumor recurrence, and long-term survival in HCC patients. snRNA RNU5E-1 may be a useful biomarker to predict the diagnosis and prognosis of HCC patients (32).

As the most prevalent internal modification of RNA epitranscriptomes in eukaryotes, m6A plays a pivotal role in regulating mRNA transcription, splicing and translation, and the biological function of snRNA (14, 23, 33, 34). Several studies suggested that m6A-mediated modifications may have important implications for HCC pathogenesis and drug response (35, 36). For example, Lin Z et al. found that the depletion of m6A writer METTL3 promoted sorafenib resistance in HCC by reducing METTL3-mediated FOXO3 mRNA stability, while the overexpression of FOXO3 induced m6A-dependent sorafenib sensitivity by suppressing autophagy in HCC (37). These results indicated m6A methylation may be an important therapeutic target for addressing sorafenib resistance in HCC patients. As stated above, both m6A and snRNAs are important regulators of HCC tumorigenesis.

Due to the role of m6A-associated snRNAs in HCC, we constructed an independent risk model based on m6A-associated snRNAs. Our results revealed that the m6A-associated snRNA model could be used as an independent risk predictor for the OS of HCC patients, and high-risk HCC patients had poorer prognosis than low-risk HCC patients. Besides, the model was superior to conventional clinicopathologic characteristics in predicting survival in HCC patients. For example, TNM staging is a crucial factor in predicting the prognosis of HCC patients. However, HCC patients of the same TNM staging may have apparently distinct prognosis, suggesting that the present prediction methods may be inaccurate. In our study, the concordance index and AUC of m6A-associated snRNA risk scores were superior to other clinicopathologic characteristics, suggesting that the risk model was independently accurate for prognostic prediction in HCC patients.

TMB refers to the number of somatic mutations in tumor genomes, which may induce the emergence of neoantigens to elicit an immune response (38, 39). Recent studies revealed that the TMB could predict prognosis and immune responses in HCC patients (39). Our study indicated that low-TMB and low-risk HCC patients obtained better OS than high-TMB and high-risk HCC patients. In addition, the TIDE prediction score could also predict the effect of immunotherapy on a variety of cancers (40, 41).. In our study, the predictions of the TIDE algorithm suggested that high-risk HCC patients had a better response to immunotherapy. Therefore, we inferred that the m6A-associated snRNA model may provide efficacy prediction for HCC immunotherapy.

Our study also provides new insights into the potential regulatory mechanisms of m6A-associated snRNAs in HCC. GO enrichment analysis revealed that m6A-associated snRNAs played a vital role in the pathogenesis of HCC through biological processes, such as serine hydrolase activity and collagen metabolism. Previous studies showed that U1 snRNA chimeric ribozymes could inhibit the synthesis of collagen I and reduce deposition of collagen I in hepatic stellate cells (HSCs), thus alleviating HSC activation and suppressing hepatic fibrosis and carcinogenesis (42). Iwai et al. found that HCV nonstructural protein 3 within serine hydrolase catalytic domain in its N-terminal region could interact with Sm-D1 component of snRNP complexes, thus affecting post-translational pathway and HCV-induced hepatocarcinogenesis process (43). In addition, m6A-associated snRNAs had regulatory effects on some biological activities and functions of immune cells, such as type II IFN response, APC co-stimulation, and cytolytic activity. Sadik et al. found that U1-snRNA could induce the expression of IFN-β, and had an anti-inflammatory potential and contributed to apoptosis and efferocytosis (44). However, the exact regulatory mechanisms of m6A-associated snRNAs in these biological processes remain unclear. For example, do m6A-associated snRNAs act as oncogenes or tumor suppressors, and can they induce immune-mediated hepatocarcinogenesis? How do m6A-associated snRNAs interact with m6A regulators, and how do these interactions affect immune responses? In the future, further studies are warranted to clarify the comprehensive molecular mechanisms of m6A-associated snRNAs in the pathogenesis of HCC.

## Conclusions

Our study suggested that m6A-associated snRNAs may be useful biomarkers for the prognosis of HCC and that the m6A-associated snRNA model could predict the effect of immunotherapy in HCC patients. Our study provided new clues to the potential biological mechanism of m6A-associated snRNAs in HCC.

## Data availability statement

The original contributions presented in the study are included in the article/**Supplementary Material**. Further inquiries can be directed to the corresponding authors.

## Author contributions

CZ, WZ, SD, and QW conceived and designed the research. CZ, YS, PL, and ZT analyzed the data. All authors made substantial contributions towards drafting the manuscript, reviewed the final manuscript for intellectual content, and authorized the submission. All authors contributed to the article and approved the submitted version.

## Fundings

This work was supported by the grants from the National Natural Science Foundation of China (82073332), the Natural Science Foundation of Zhejiang Province (LQ19H030002), the Medical Science and Technology Project of Zhejiang Province (2023KY182), and the Youth Fund of the Affiliated Hospital of Hangzhou Normal University (2021YN2021027).

## Conflict of interest

The authors declare that the research was conducted in the absence of any commercial or financial relationships that could be construed as a potential conflict of interest.

## Publisher's note

All claims expressed in this article are solely those of the authors and do not necessarily represent those of their affiliated organizations, or those of the publisher, the editors and the reviewers. Any product that may be evaluated in this article, or claim that may be made by its manufacturer, is not guaranteed or endorsed by the publisher.

## Supplementary material

Supplementary Figure 1 Kaplan-Meier curve analysis of OS in female low- and high-risk HCC patients on clinicopathological characteristics (including age, grade, and TNM staging).

Supplementary Figure 2 Kaplan-Meier curve analysis of OS in male low- and high-risk HCC patients on clinicopathological characteristics (including age, grade, and TNM staging).
