# Supplementary figures and images for: Implications of m6A-associated snRNAs in the prognosis and immunotherapeutic responses of hepatocellular carcinoma
Source: Front Immunol. 2022 Nov 2;13:1001506. doi: 10.3389/fimmu.2022.1001506 (PMC9667552; doi:10.3389/fimmu.2022.1001506)

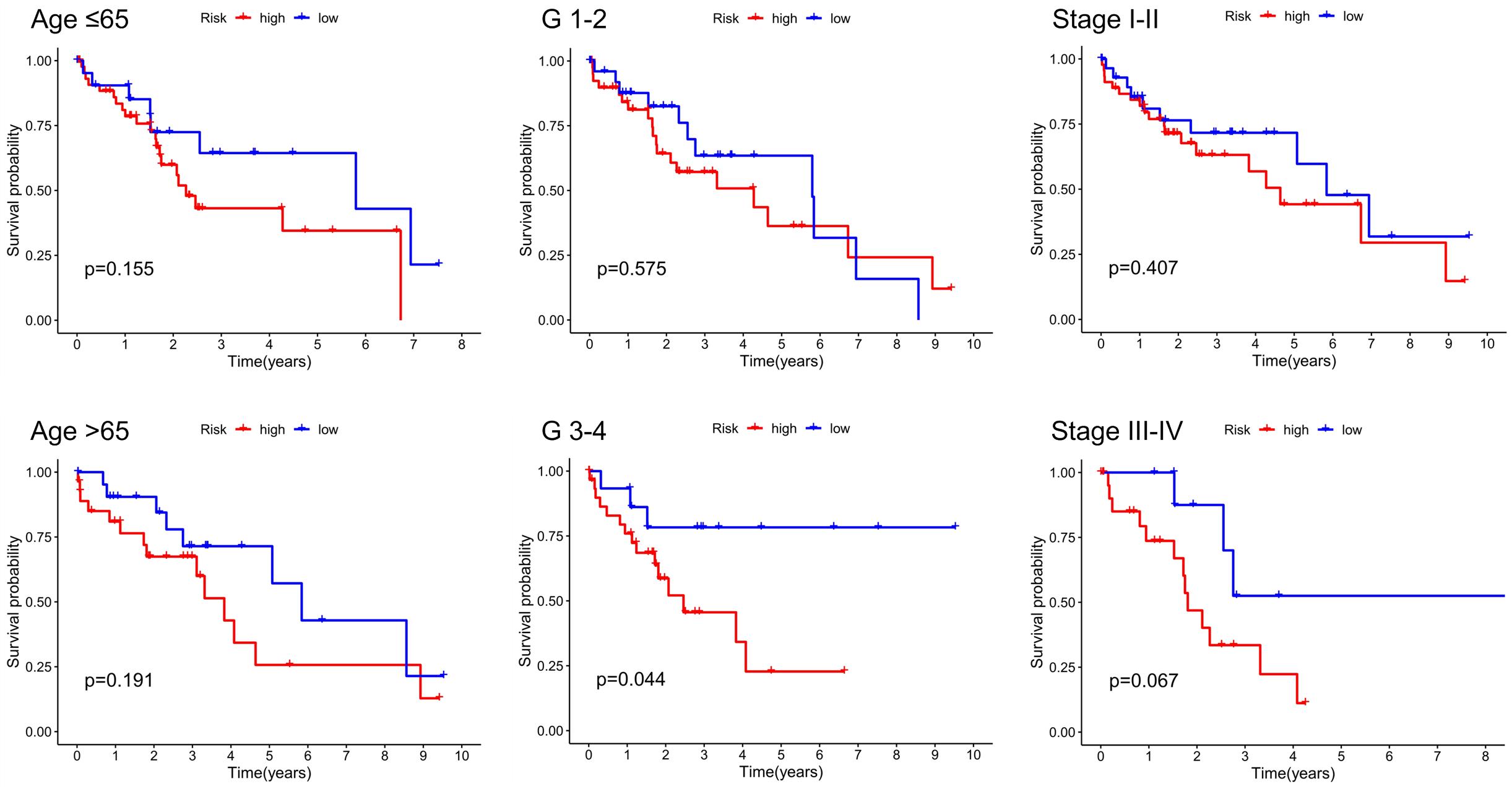

Supplement: Supplementary Figure 1 — Kaplan-Meier curve analysis of OS in female low- and high-risk HCC patients on clinicopathological characteristics (including age, grade, and TNM staging). [file Image_1.jpeg]

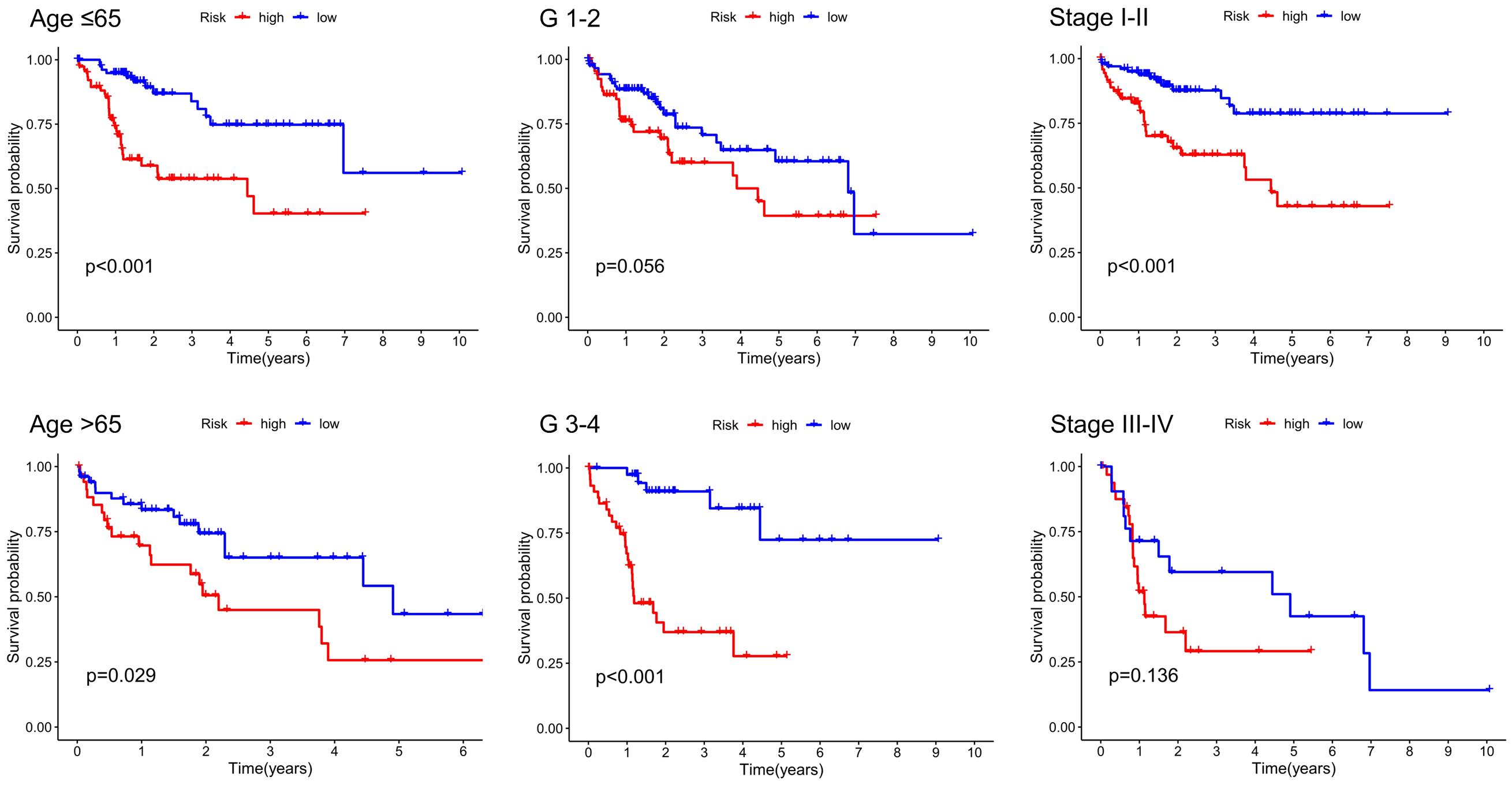

Supplement: Supplementary Figure 2 — Kaplan-Meier curve analysis of OS in male low- and high-risk HCC patients on clinicopathological characteristics (including age, grade, and TNM staging). [file Image_2.jpeg]
